# miRNA Expression Profile and Involvement of Let-7d-APP in Aged Rats with Isoflurane-Induced Learning and Memory Impairment

**DOI:** 10.1371/journal.pone.0119336

**Published:** 2015-03-23

**Authors:** Ting Luo, Shuzhou Yin, Rong Shi, Chengshi Xu, Yun Wang, Jun Cai, Yun Yue, Anshi Wu

**Affiliations:** 1 Department of Anesthesiology, Chaoyang Hospital, Capital Medical University, Beijing, China; 2 Suzhou Kowloon Hospital, Shanghai JiaoTong University School of Medicine, Suzhou, Jiangsu, China; 3 Department of Anesthesiology, Beijing Jishuitan Hospital, Fourth Medical College of Peking University, Beijing, China; 4 Department of Cardiology, Chaoyang Hospital, Capital Medical University, Beijing, China; University of Virginia, UNITED STATES

## Abstract

MicroRNAs (miRNAs) play a key role in different nervous system diseases. We sought to determine the role of miRNAs in isoflurane-induced learning and memory impairment in aged rats. Male Sprague-Dawley (SD) rats of 18 month were randomly assigned to control group (exposed to mock anesthesia), 2-hour group and 6-hour group (exposed to 2% isoflurane for 2 and 6 hours respectively). By Morris Water Maze, 6-hour group showed impaired learning and memory ability while 2-hour group not. As shown by miRNA array, control group and 2-hour group showed a similar miRNA expression profile. And 38 miRNAs are differently expressed in 6-hour group compared to the other 2 groups, including 21 up-regulated miRNAs and 17 down-regulated miRNAs. And 4 of the differentially expressed miRNAs were validated independently by qRT-PCR. Let-7d was downregulated in 6-hour group. Additionally, we demonstrated that amyloid precursor protein (APP) was a direct target of let-7d by Fluorescent report assay. Increased expression of APP and amyloid-β (Aβ) were found in the hippocampi of 6-hour group. Downregulation of let-7d might contribute to isoflurane-induced learning and memory impairment through upregulating its target APP, and increasing the production of Aβ subsequently.

## Introduction

Isoflurane is one of the most common general anesthetics and has been widely used in recent years. However, emerging evidence demonstrates that isoflurane could increase apoptosis of neurons, reduce neurogenesis, and change the ultrastructure of synapse, subseuquently producing learning and memory impairment[1, 2, 3, 4, 5]. Aged neurons are especially vulnerable to isoflurane[6]. Along with increased aging population, it’s very important to understand the underlying molecular mechanisms of isoflurane-induced learning and memory impairment in the elderly.

MicroRNAs (miRNAs) are a group of 20-nt-long, endogenous, non-coding RNAs that regulate protein expression at the post-transcriptional level through direct binding to the 3′ untranslated regions (UTRs) of their target mRNAs[7]. Hundreds of miRNAs have been identified in the mammalian central nervous system (CNS) and are reported to have critical roles in the development of CNS, neurodegenerative disorders and nervous system neoplasm[8, 9, 10]. However, the role of miRNAs in isoflurane-induced learning and memory disability of aged rats is largely unknown.

In this study, we examined miRNA expression patterns in the hippocampi of control group (n = 16, exposed to mock anesthesia), 2-hour group (n = 16, exposed to 2% isoflurane for 2 hours) and 6-hour group (n = 16, exposed to 2% isoflurane for 6 hours). Compared to the other 2 groups, 21 miRNAs were up-regulated, and 17 miRNAs were downregulated in the 6-hour group. Four of the differentially expressed miRNAs (miR-9, miR-143, miR-146a, let-7d) were validated independently in samples from these 3 groups. We further determined the amyloid precursor protein (APP) to be the direct target of let-7d by bioinformatic analysis and Fluorescent reporter assays. In addition, we revealed the increased expression of APP and Aβ levels in 6-hour group, while let-7d was downregulated. Accumulating evidence suggests that increased APP expression could increase the levels of Aβ, which is produced from APP by sequential proteolytic cleavages through two proteases, β-secretase and γ-secretase. It has also been demonstrated that Aβ can exert neurotoxic effects by inducing cell degeneration and death, synaptic dysfunction, and neurodegeneration, eventually leading to learning and memory impairment[11, 12]. The aims of this study were to determine the miRNA expression pattern and the role of let-7d-APP in aged rats with learning and memory impairment induced by isoflurane.

## Materials and Methods

### Animals and grouping

This protocol was approved by the Standing Committee on Animals at Capital Medical University. 48 male Sprague-Dawley (18-month-old) rats, weighing 450–550g, were acquired from the Institute of Experimental Animal of Medical Scientific Academy in Sichuan, P. R. China. After 1-week acclimation period in the laboratory, rats were randomly assigned into 3 groups, control group (n = 16, exposed to mock anesthesia), 2-hour group (n = 16, exposed to 2% isoflurane for 2 hours), and 6-hour group (n = 16, exposed to 2% isoflurane for 6 hours). All animals were treated according to the standards for ethical treatment of laboratory animals as published by the National Institutes of Health. And efforts were made to minimize the number of animals used. All rats were sacrificed after reversal testing, which is performed on the 3rd day after anesthesia.

### Isoflurane anesthesia

2-hour group and 6-hour group received 2% isoflurane (NO26C818A, Baxter, America) for 2 and 6 hours respectively (anethesia machine: Sulla909V, Dragger, Germany), whereas the control group (16 18-month-old rats) received air/oxygen at identical flow rates in identical chambers. The isoflurane concentration in the chamber was monitored with a vaporizer. The rectal temperature was maintained at 37.0±0.5°C. Animals were visually inspected for respiratory effort and skin color. Pulse oximeter oxygen saturation (SpO2) were routinely monitored during anesthesia. Mean arterial blood pressure (MAP) was recorded by using a non-invasive sphygmomanometers (ZH-HX-Z, Anhui province, China). According to previous study[13], left ventricle blood was collected from 4 rats in each group, and blood gases were measured at the end of anesthesia with an arterial blood gas analyzer (ABL 725, Radiometer A/S, Copenhagen, Denmark).

### Learning and memory testing

Before and after anesthetic exposures, learning and memory testing of rats was performed in the Morris Water Maze (MWM). MWM is 150cm in diameter and was filled with opacified water (22±1°C) to the height of 1.5cm above the top of the movable 15cm diameter platform. Rats were tracked with a video camera mounted above the pool that fed to a computer running IMAQ PCI-1407 software. The time between trilas was at least 60 min. Reference meomory testing was performed for 5 days (4 trials×2 per day) before anesthesia. According to previous study[14], reversal testing was performed on the 3^rd^ day after anesthesia.

Reference memory testing (RMT)—For all trials, the platform was placed in the target quadrant and the rats started in a random quadrant. The maximum swimming time was 120 seconds, if rats failed to find the hidden platform in 120s, they would be guided to the platform gently and remain on the platform for 10–15s. The time to reach the platform (Escape latency periods, ELP) and the swim speed were recorded for each trial.

Reversal testing—The platform was moved to the opposite quadrant of the pool but all distal visual cues remained consistent. Preceding the start of the test, animals were placed on the platform for 30s and then swam in the pool to locate the platform in the new target quadrant for 3 trials. The maximum swimming time was set to be 120s. ELP and the swim speed were recorded for each trial. Reversal learning could measure how quickly an animal is able to extinguish their initial learning of the platform’s position and acquire a direct path to the new location[15].

### RNA purification, labeling, miRNA array and data analysis

Total RNA of hippocampus was extracted with TRIzol (Invitrogen) and an Rnase mini kit (QIAGEN) in accordance with the manufacturer’s instruction. The quality of the total RNA was verified by an Agilent 2100 Bioanalyzer profile. Then total RNA was labeled using a miRCURY Hy3/Hy5 power labeling kit (Exiqon, Denmark).The miRNA expression profiling of hippocampus from 4 rats in each group was performed using miRCURY LNA Array (v.14.0) system. The array sequence contents were sourced from the Sange miRBASE database (Release 14.0). Scanning was performed with an Axon GrenePix 4000B microarray scanner. The raw intensity of the image was read by the GenePix pro V6.0. Normalized log2-transformation were applied. System-related variations was eliminated by a filter on low miRNAs expression. Differentially expressed miRNAs were identified as having a more than 1.5-fold expression difference among groups, with a statistically significant *p* value < 0.05 calculated by ANOVA statistic.

### Real-time quantitative reverse transcription-PCR

We found that miR-146a, miR-9, miR-143 and let-7d might be connected with learning and memory ability through review of the literature and prediction of the target gene[16,17,18]. In addition, the four miRNAs were found to be expressed with significant difference among three groups. Hence these 4 differentially expressed miRNAs were measured with quantitative reverse transcription (RT) PCR to verify the findings of miRNAs array. A total of 5ug RNA was reverse trascribed to cDNA by utilizing MMLV reverse transcriptase (Epicentre, Madison, WI) according to the manufacturer’s instruction. Specific primers were designed through using the Primer Express software (Applied Biosystems, USA). In presence of SYBR 1 Green (BioFlux, Japanese), quantitative PCR was performed by an ABI PRISM7500 system (Applied Biosystems, Foster Cit, CA). The relative abundance of miRNAs was normalized by the expression level of U6 RNA.

### Fluorescent Reporter Assays

In order to confirm the APP was the target of let-7d, Fluorescent reporter experiments were conducted. The 3’UTR and mut-3’UTR from the APP gene was cloned into a vector containing the enhanced green fluorescent protein (EGFP) cDNA (pSGG_3UTR; Swithc-Gear Genomics). And the resulted vector were introduced into 293T cells together with the pcDNA3.1-pri-let-7d plasmid or control plasmid (pcDNA3.1) (Dharmacon, Lafayette, CO, USA). Cells were lysed 48h after transfection, and EGFP intensity was measured with a Fluorescence Spectrophotometer F-4500 (Hitachi, Tokyo, Japan) and normalized by the amount of total protein, which was quantitated by Coomassie bright blue G-250 stain.

### Western-blot

Total protein from hippocampi was extracted with protein lysis buffer (20mmol/l Tris-HCL, pH 7.6, 150mmol/l NaCl, 1% NP-40) containing protease inhibitor cocktail. Lysates were resolved by SDS-polyacrylamide gel electrophoresis and electrotransferred to PVDF membranes (Bio-Rad, Hercules, CA). The blots were incubated with rabbit anti APP (Abcam Inc, Cambridge, MA) and anti-GAPDH (Santa Cruz Biotechnology, Santa Cruz, CA) overnight at 4°C. And then the blots were incubated with secondary antibodies conjugated with horseradish peroxidase (Santa Cruz Biotechnology; 1:3500 dilution) for 2h while shaking at room temperature. The signals were detected with enhanced chemiluminescence (Amersham Pharmacia Biotech).

### Statistical Analysis

The statistical analysis was accomplished with SPSS 13.0 for windows. Values are expressed as means ±SD. The normal distribution of data was tested by the 1-sample Kolmogorov-Smirnov test. A One-Way analysis of variance (ANOVA), followed by a LSD (Least Significant Difference) test were used to compare the measurement data of 3 groups, Statistical significance was set as p < 0.05.

## Results

### Mean arterial pressure and blood gas

Mean arterial pressure (MAP) was similar in the three groups and remained within the physiological range. Blood gas analysis at the end of anesthesia revealed no differences between the three groups (Table 1).

**Table 1:** Comparison of physiological parameters between 3 groups (n = 4, mean±s).

Groups	MAP	pH	pCO2	pO2	Glucose
	(mmHg)		(mmHg)	(mmHg)	(mmol/L)
Control	104±2	7.36±0.09	53±10	99±1	5.06±0.65
2-hour	102±3	7.34±0.03	52±5	98±3	4.27±1.13
6-hour	99±4	7.31±0.05	58±7	96±7	4.45±0.79

### Learning and memory testing

**Fig 1:**
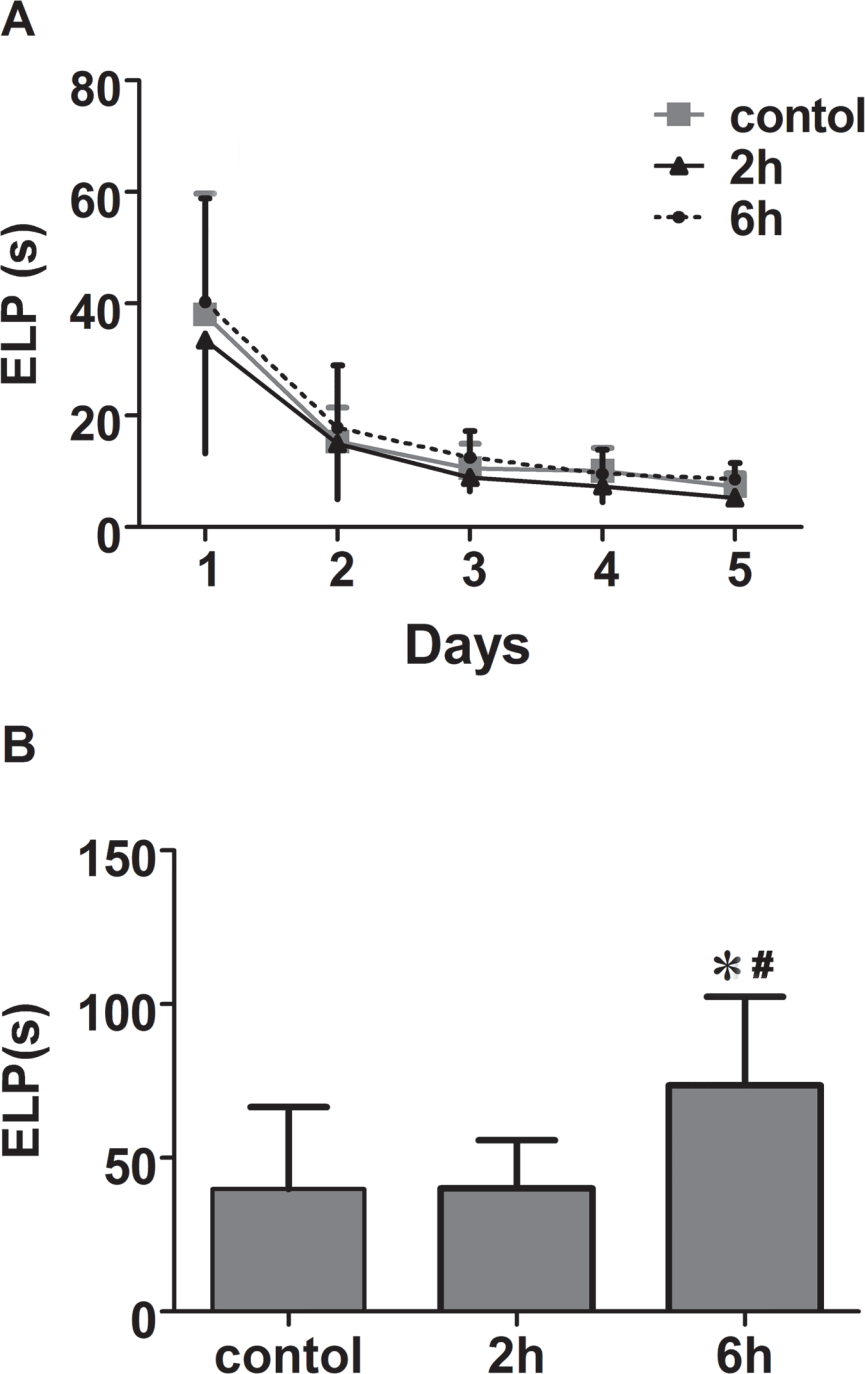
Learning and memory testing of 3 groups before and after anesthesia. **A**, The time to reach the platform (Escape latency period, ELP) for 3 groups before anesthesia (Reference memory testing). No difference was found among groups in the reference memory testing before anesthesia exposure. **B**, Escape latency period of 3 groups after anesthesia (Reversal Testing). After exposure to the isoflurane or mocked anesthesia, 6-hour group took longer to reach the platform during the reversal testing than the other 2 groups, while escape latencies of 2-hour group were not longer than contol group. *p<0.05 vs. control, **#**p<0.05 vs. 2-hour group, n = 16 per group.

Prior to the anesthetic exposures, reference memory testing showed no between-group differences in escape latency periods (Fig. 1A). After exposure to the isoflurane or mock anesthesia, 6-hour group took longer to reach the platform during the reversal testing than 2-hour group (*p* < 0.05) and control group (*p* < 0.05), there was no significant difference between the escape latency periods of control group and 2-hour group(Fig. 1B).

### Microarray analysis of 3 groups

**Fig 2:**
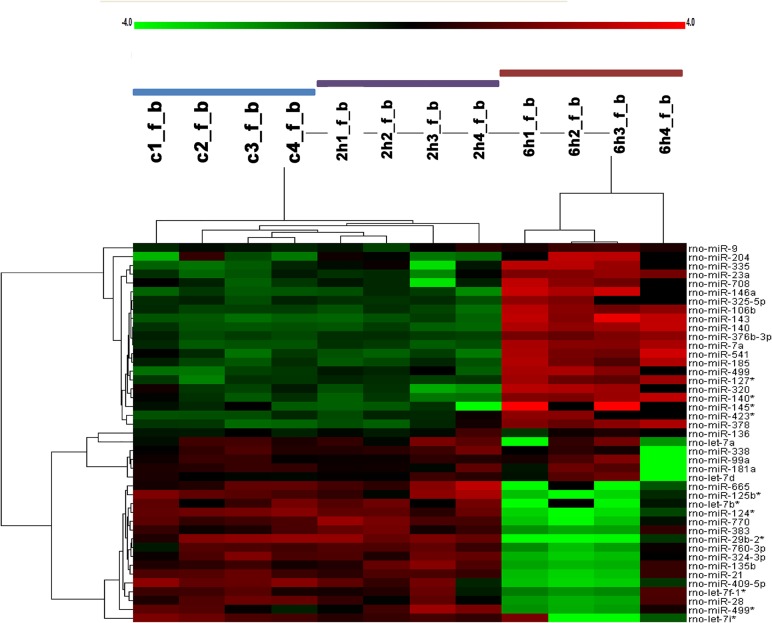
Heat map of microRNA microarray expression data from hippocampus samples of 3 groups. The expression of miRNAs is hierarchically clustered on the y axis, and hippocampus samples of 3 groups are hierarchically clustered on the x axis. Number with C represent control group, number with 2h represent 2-hour group, number with 6h means 6-hour group. The legend on the right indicates the miRNA represented in the corresponding row. The color scale on the right show the relative miRNA expression, green means downregulation, while red means upregulation, n = 4 per group.

The intersection of differently expressed miRNAs among 3 groups is shown in Fig. 2. As illustrated by it, control group and 2-hour group show a similar miRNA expression profile. Compared to the other 2 groups, 21 miRNAs are upregulated in 6-hour group as shown in the upper portion of Fig. 2, miR-9, miR-204, miR-335, miR-23a, miR-708, miR-146a, miR-325-5p, miR-106b, miR-143, miR-140, miR-376b-3p, miR-7a, miR-541, miR-185, miR-499, miR-127*, miR-320, miR-140*, miR-145*, miR-423*, miR-378. And 17 miRNAs are downregulated as shown in the lower part of this figure, let-7d, miR-665, miR-125b*, let-7b*, miR-124*, miR-770, miR-383, miR-29b-2*, miR-760-3p, miR-324-3p, miR-135b, miR-21, miR-409-5p, let-7f-1*, miR-28, miR-499*,let-7i* (Table 2).

**Table 2:** Differentially expressed miRNAs in 6-hour group compared with the other 2 groups.

Upregulated miRNAs	Downregulated miRNAs
miR-9	let-7d
miR-204	miR-665
miR-335	miR-125b
miR-23a	let-7b
miR-708	miR-124
miR-146a	miR-770
miR-325-5p	miR-383
miR-106b	miR-29b-2
miR-143	miR-760-3p
miR-140	miR-324-3p
miR-376b-3p	miR-135b
miR-7a	miR-21
miR-541	miR-409-5p
miR-185	let-7f-1
miR-499	miR-28
miR-127	miR-499
miR-320	let-7i
miR-140	
miR-145	
miR-423	
miR-378	

### qPCR validation of selected miRNAs

**Fig 3:**
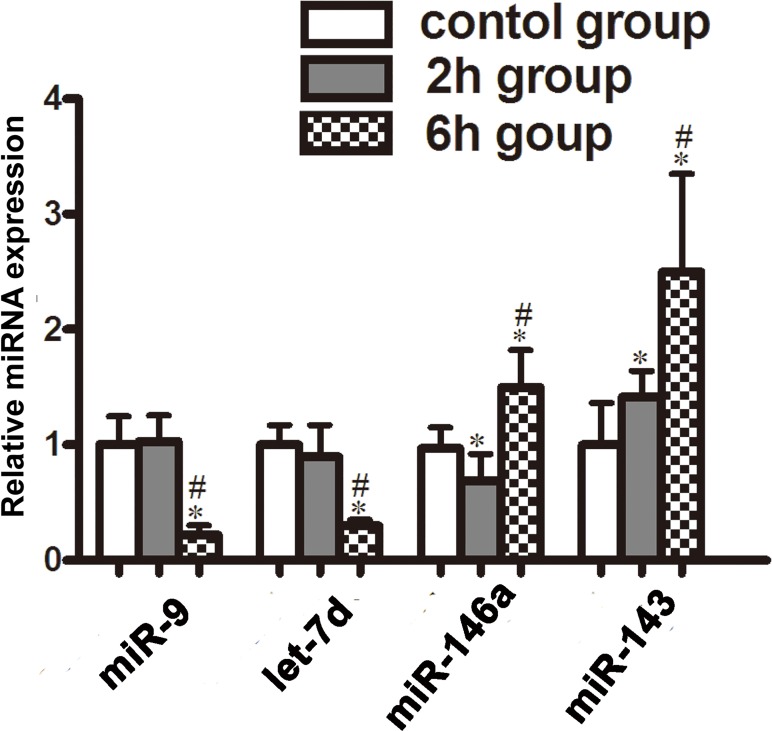
Validation of microRNA (miRNA) microarray data by quantitative reverse-transcription polymerase chain reaction. The relative miRNAs expression was calculated in relation to that in control group. Compared to the other 2 groups, there was a significant decrease in miR-9, let-7d and a trend of increase in miR-146a, miR-143 in 6-hour group. *p<0.05 vs. control, **#**p<0.05 vs. 2-hour group, n = 8 per group.

Four of the differentially expressed miRNAs (miR-9, miR-143, miR-146a, and let-7d) were selected for qPCR validation. Table 3 shows the reverse transcription primers and qPCR primers for each miRNA. The relative expressions of the four miRNAs were normalized to the expression of the internal control (U6). The measurement data of 3 groups were compared with One-Way ANOVA, and the comparison between 2 groups was performed with LSD. Fig. 3 shows the relative expression of each miRNA in these 3 groups. Our results demonstrated that compared to the other 2 groups, there was a significant decrease in miR-9, let-7d in 6-hour group, while there was a trend of increase in miR-146a and miR-143 (*p* < 0.05 for each). Although miRNA profiling showed an increase miR-9 expression in 6-hour group (Fig. 2), our quantitative qPCR assay showed that the expression of miR-9 was downregulated in this group (Fig. 3).

**Table 3:** Sequences of the primers used in the SYBR-green-based quantitative RT-PCR validation.

Primer name	Primer sequence
rno-miR-9 RT	5′GCGCGTGAGCAGGCTGGAGAAATTAACCACGCGCTCATAC3′
rno-miR-9 F	5′CCTCTTTGGTTATCTAGC3′
rno-miR-146 RT	5′GCGCGTGAGCAGGCTGGAGAAATTAACCACGCGCAACCCA3′
rno-Let-146 F	5′CTGAGAACTGAATTCCA3′
rno-miR-143 RT	5′GCGCGTGAGCAGGCTGGAGAAATTAACCACGCGCTGAGCT3′
rno-miR-143 F	5′CTGAGATGAAGCACTGTA3′
rno-let-7d RT	5′GCGCGTGAGCAGGCTGGAGAAATTAACCACGCGCAACTAT3′
rno-let-7d F	5′CAGAGGTAGTAGGTTGC3′

### APP is a direct target of let-7d

**Fig 4:**
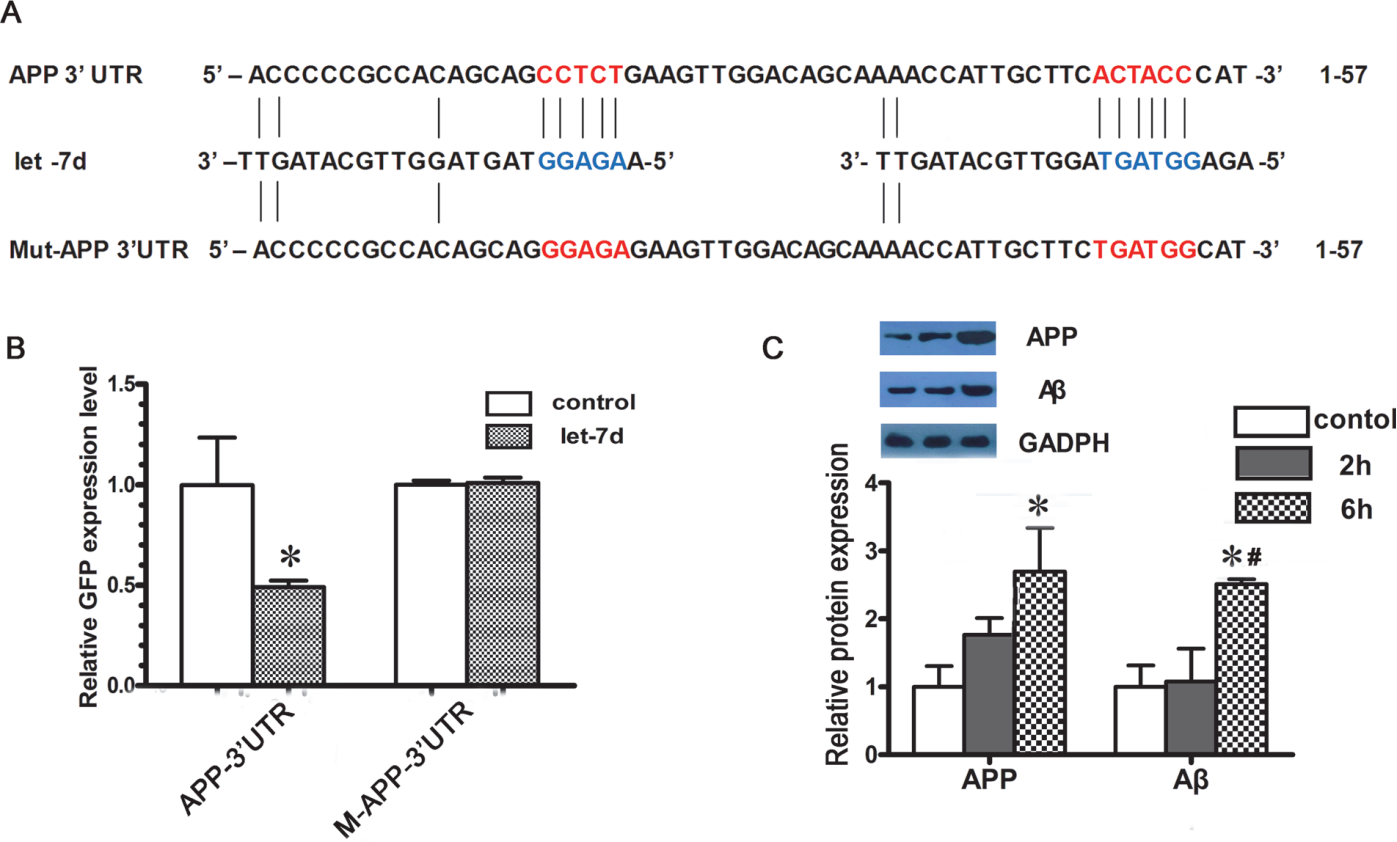
APP is a direct target gene of let-7d. **A**, The predicted duplex of let-7d, and its target site in the untranslated region (UTR) of APP, as well as the mutations made in the APP 3’UTR. **B**, The EGFP expression of reporter vector containing the APP 3’UTR in 293T cells was downregulated by let-7d, and that of the reporter vector containing the mutated APP 3’UTR was not suppressed. *p<0.05, n = 4 per group. **C**, The expression of APP and Aβ in 3 groups. Compared to the other 2 groups, the expression level of APP and Aβ was significantly increased in 6-hour group. GAPDH expression was used as a loading control. The expression of APP and Aβ was normalized by the expression of the GAPHD. The relative APP and Aβ expression was calculated in relation to that in control group. The experiments had been repeated four times and the results were similar to the one shown. *p<0.05 vs. control, **#**p<0.05 vs. 2-hour group, n = 4 per group.

Computational predictions by TargetScan, miRanda, and PicTar indicated that let-7d could target APP. To confirm that APP was the direct target of let-7d, we co-transfected 293T cells with pcDNA3.1-pri-let-7d or control plasmid along with the EGFP reporter vector containing the APP 3′ UTR. Our results demonstrated more than 50% lower EGFP expression in the APP 3′ UTR group when co-transfected with pri-let-7d (*P* < 0.01, Fig. 4A and 4B). To further verify the specificity of the miRNA-mediated repression, we mutated the let-7d binding site in the 3’ UTR of APP. As a result, let-7d could no longer suppress EGFP expression (Fig. 4A and 4B). Thus, we can conclude that APP is a direct target of let-7d.

### Expression of APP and Aβ protein in 3 groups

The downregulation of let-7d in 6-hour group suggested that expressions of APP and Aβ might be upregulated in this group. Western-blot was used to compare the expressions of APP and Aβ protein of 3 groups. The results showed increased APP and Aβ in 6-hour group (*p* < 0.05) (Fig. 4C).

## Discussion

The commonly used anesthetic isoflurane is potentially neurotoxic, with previous studies proving that inhaling isoflurane induces dose- and time-dependent damage to the nervous system, including hippocampal slices, neurosecretory PC12 cells, and primary cortical and striatal neurons[1, 19, 20, 21]. In animal studies, isoflurane exposure could cause widespread neurodegeneration and subsequent memory impairement[4]. The present study demonstrated that 6 hours of exposure to isoflurane impaired learning and memory abilities of aged rats, while 2 hours did not.

Increasing evidence shows that a majority of miRNAs are expressed in the brain in a spatially and temporally controlled manner[22], and play important roles in brain development and function, as well as in nervous system diseases [23, 24, 25]. Hence, miRNAs could potentially serve as biomarkers and targets for therapy for nervous system diseases. And it’s meaningful to identify the role of miRNAs in isoflurane induced learning and memory impairment, which could lead to discovering new targets for treatment. Hippocampi have a close relationship with learning and memory. By comparing their miRNA expression profile, we defined a distinct hippocampal miRNA expression profile in rats with isoflurane-induced learning and memory impairment. 38 miRNAs were found to be differentially expressed in 6-hour group compared with the other 2 groups, 21 miRNAs were increased and 17 miRNAs were decreased in this group with learning and memory impairment.

Our results showed that let-7d miRNA was downregulated in the rats with isoflurane-induced learning and memory impairment. According to the prediction of miRNA targets, let-7d could regulate 1072 conserved targets in human, and 864 conserved targets in rats (http://www.targetscan.org). Furthermore, we found that amyloid precursor protein (APP) was a direct target of let-7d. And our Western-blot data showed that the expression levels of APP and Aβ was upregulated in the impaired rats. Collectively, these data support a potential role of let-7d in the development of learning and memory impairment of aged rats caused by isoflurane. It’s plausible that by upregulating the levels of APP, and the production of Aβ consequently, the downregulation of let-7d is involved in learning and memory impairment caused by isoflurane. Previous in vitro studies have shown that isoflurane could promote Aβ peptide oligomeriation and cytotoxicity in rat pheochromocytoma cells and H4 human neuroglioma cells[26, 27]. Our results are in agreement with these studies, and further help to reveal the question that how isoflurane promotes Aβ production. Based on the current data, we could not completely rule out the possibility that parallel mechanisms affected by let-7d is involved in isoflurane-induced learning and memory impairment. In addition, whether the downregulation of let-7d is sufficient to cause learning and memory impairment, still need further investigation. And another limitation of the present study is that previous studies have suggested that inhalational anesthetics may have different potency to cause neurotoxicity[28]. Thus the possible differential potency of anesthetics on miRNA expression need to be tested in further study.

In conclusion, the present results provide the first evidence that isoflurane-induced learning and memory impairment in aged rats is associated with let-7d downregulation, which apparently increases expression of its target gene APP, consequently upregulating the expression of Aβ. This finding provides an important and novel perspective for decoding the complex mechanism of miRNA/mRNA interplay during this process. However, further experiments using let-7d knockout and transgenic animal models are needed for validation of the treatment role of let-7d in isoflurane-induced learning and memory impairment in aged rats. Let-7d has been demonstrated to directly regulate multiple cell proliferation genes, such as RAS and c-Myc, thus controlling cell proliferation and apoptosis [29]. Since decreased neurogenesis and increased neural apoptosis play important roles in learning and memory disability, it’s possible that let-7d downregulation could cause learning and memory impairment by activating other pathways. Further studies are needed to explore the mechanism of let-7d involvement in the process of isoflurane-induced learning and memory impairment.

## Disclosure statement

All animals used in this study was approved by the Standing Committee on Animals at Capital Medical University.
